# Oxidized Melanoma Antigens Promote Activation and Proliferation of Cytotoxic T‐Cell Subpopulations

**DOI:** 10.1002/advs.202404131

**Published:** 2024-07-03

**Authors:** Ramona Clemen, Lea Miebach, Debora Singer, Eric Freund, Thomas von Woedtke, Klaus‐Dieter Weltmann, Sander Bekeschus

**Affiliations:** ^1^ ZIK plasmatis Leibniz Institute for Plasma Science and Technology (INP) Felix‐Hausdorff‐Str. 2 17489 Greifswald Germany; ^2^ Department of Dermatology and Venerology Rostock University Medical Center Strempelstr. 13 18057 Rostock Germany; ^3^ Department of Neurosurgery Wien University Medical Center Vienna 1090 Austria; ^4^ Institute for Hygiene and Environmental Medicine Greifswald University Medical Center Ferdinand‐Sauerbruch‐Str. 17475 Greifswald Germany

**Keywords:** CAP, gas plasma technology, neoantigens, oxPTM, plasma medicine, reactive oxygen species (ROS), tumor‐associated antigens

## Abstract

Increasing evidence suggests the role of reactive oxygen and nitrogen species (RONS) in regulating antitumor immune effects and immunosuppression. RONS modify biomolecules and induce oxidative post‐translational modifications (oxPTM) on proteins that can alarm phagocytes. However, it is unclear if and how protein oxidation by technical means could be a strategy to foster antitumor immunity and therapy. To this end, cold gas plasma technology producing various RONS simultaneously to oxidize the two melanoma‐associated antigens MART and PMEL is utilized. Cold plasma‐oxidized MART (oxMART) and PMEL (oxPMEL) are heavily decorated with oxPTMs as determined by mass spectrometry. Immunization with oxidized MART or PMEL vaccines prior to challenge with viable melanoma cells correlated with significant changes in cytokine secretion and altered T‐cell differentiation of tumor‐infiltrated leukocytes (TILs). oxMART promoted the activity of cytotoxic central memory T‐cells, while oxPMEL led to increased proliferation of cytotoxic effector T‐cells. Similar T‐cell results are observed after incubating splenocytes of tumor‐bearing mice with B16F10 melanoma cells. This study, for the first time, provides evidence of the importance of oxidative modifications of two melanoma‐associated antigens in eliciting anticancer immunity.

## Graphical abstract

Reactive oxygen and nitrogen species (ROS) modify biomolecules and induce oxidative post‐translational modifications (oxPTM), leading to altered immune cell response and antitumor immune effects. Oxidation of melanoma‐associated antigens MART (oxMART) and PMEL (oxPMEL) correlate with significant changes in activity, proliferation, and CD8^+^ T‐cell differentiation. Oxidized tumor antigens further altered cytokine secretion of tumor‐infiltrated leukocytes.

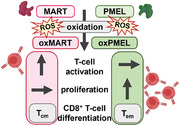

## 1 Introduction

Malignant, metastatic melanoma is the most dangerous form of all skin cancers, and therapeutic failure and the lack of universal therapy are owed to melanoma heterogeneity and a high degree of mutational burden.^[^ ^1^, ^2^, ^3^ ^]^ Excessive prevalence of somatic mutation in melanoma can lead to the generation of tumor‐associated antigens (TAA) and neoantigens, which can be recognized by adaptive immunity that can initiate endogenous tumor cell targeting.^[^ ^4^, ^5^ ^]^ Melanoma‐specific antigens that bind to molecules of the major histocompatibility complex (MHC).^[^ ^6^ ^]^ were identified decades ago, such as peptides of the proteins MAGE1 (Melanoma Antigen Gen), MAGE3, Pmel17 (Premelanosome Protein, origin of gp100 peptide), Melan A (in humans), MART (Melanoma Antigen Recognized by T‐cells), and PMEL (in mice). In humans, the most prominent lymphocyte responses are directed against tumor antigens MART and gp100,^[^ ^7^ ^]^ making these TAAs important objects of investigation and targets to treat. Different antigen‐specific T‐cells that recognize their specific antigen through the interaction between the (T‐cell receptor) TCR and the loaded MHC complex are important for tumor killing and characteristic of the adaptive immune system. An adaptive immune response is usually initiated by an innate immune response, starting with an inflammatory response. Herein, RONS such as hydrogen peroxide (H_2_O_2_), hypochlorous acid (HOCl), and nitric oxide (NO) are naturally produced to act as signaling molecules for immune cells, such as modulating T‐cell activation and tolerance.^[^ ^8^ ^]^ Furthermore, the oxidative burst and present RONS oxidize proteins and the resulting oxidative post‐translational modifications (oxPTM), such as chlorination, oxidation, and nitration, serve as an intrinsic adjuvant and mechanistically are considered to have a function similar to damaged associated molecular patterns (DAMPs).^[^ ^9^, ^10^ ^]^ Various immunological consequences of oxPTM have been published, such as the activation of dendritic cells,^[^ ^11^, ^12^ ^]^ B‐cells,^[^ ^13^ ^]^ or T‐cells,^[^ ^14^ ^]^ and due to the immunogenicity of oxPTMs, RONS have previously been used to optimize tumor vaccines. For instance, therapeutic vaccines consisting of HOCl‐oxidized lysates (oxLys) encapsulated in polymer‐nanoparticles^[^ ^15^ ^]^ or oxLys‐loaded dendritic cell (DC) based vaccines^[^ ^16^, ^17^ ^]^ led to reduced tumor burden. In addition to chlorination through HOCl treatment, multi‐RONS treatments lead to various other modifications with other immunological consequences. Such a multi‐RONS environment can be created by medical cold plasma technology, also called cold physical plasma (CPP), cold atmospheric plasma (CAP), nonthermal plasma (NTP), or low‐temperature plasma (LTP). This gas plasma operates at body temperature and is a multi‐component system composed of light (UV photons, visible, infrared), electric fields, ions, electrons, temperature, and RONS.^[^ ^18^, ^19^ ^]^ One advantage of using cold plasma is the generation of highly reactive species that allows to study of RONS‐effects of proteins and can be modulated by changing the feed gas compositions.^[^ ^20^, ^21^, ^22^ ^]^ Furthermore, cold plasma is safe for medical applications, such as treating infected wounds or cancers, and some plasma sources are CE‐certified and accredited as medical devices (reviewed in refs. [23, 24, 25, 26]). Previously, direct treatment of tumors with cold plasma technology was shown to reduce tumor burden by increasing markers associated with immunogenic cell death and elevating the number of immune cells in the tumor tissue.^[^ ^27^, ^28^, ^29^ ^]^ Further studies revealed immunotherapeutic aspects of cold plasma therapy, such as abscopal effects in vivo.^[^ ^30^ ^]^ and increased immune cell activity ex vivo.^[^ ^31^, ^32^ ^]^ However, in these studies, cold plasma technology was applied as a direct treatment, whereas plasma can alternatively be used to investigate the effects of individual biomolecules. Tomić and colleagues fed dendritic cells (DCs) with tumor cells after multi‐RONS generated cold plasma treatment, leading to elevated activation and maturation, so they proposed plasma as a tool to optimize DC‐loading for adoptive cell transfer.^[^ ^33^ ^]^ A similar enhanced inflammatory profile was observed in our group after incubating DCs with cold plasma‐treated peritoneal carcinoma cells.^[^ ^34^ ^]^ Melanoma cell lysates treated with plasma‐generated multiple‐RONS resulted in limited tumor mass in vivo when injected as a prophylactic vaccine.^[^ ^35^, ^36^ ^]^ Oxidized whole tumor lysates contain various proteins that promote an antitumor immune response, so it is questionable whether individual oxidized melanoma proteins can achieve the same effect.

**Figure 1:**
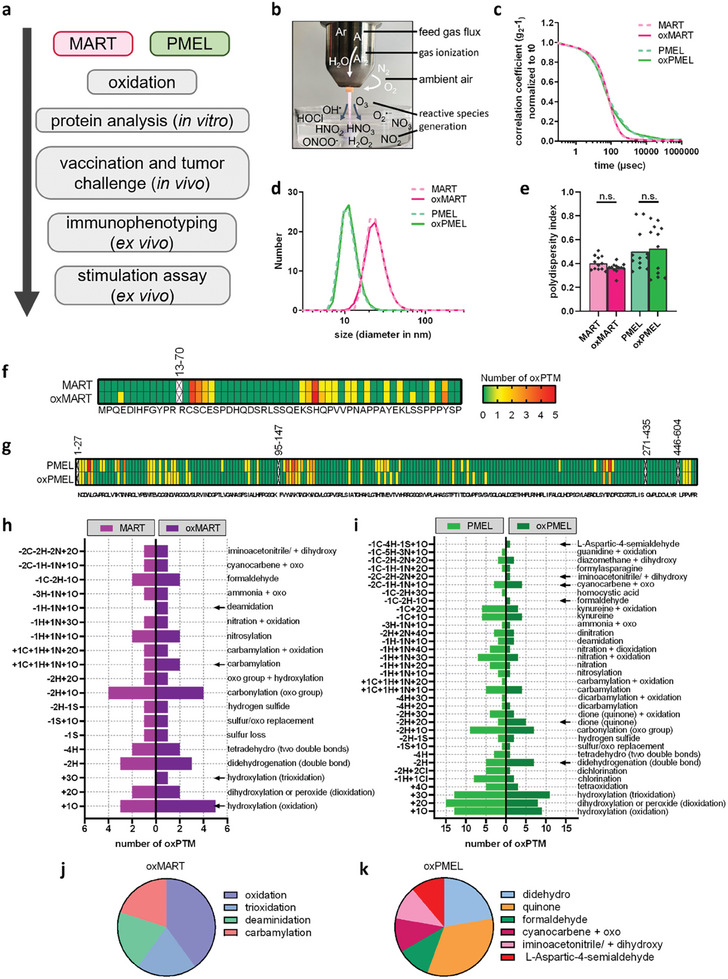
Cold plasma‐derived modifications in melanoma‐associated antigens MART and PMEL. a) study workflow; b) cold plasma‐generated reactive species; dynamic light scattering showed c) no difference of correlation coefficient and quantified area under the curve (AUC) and d) no changes of protein size or e) polydispersity index. Data are mean values that include all individual data points from four independent experiments with three technical replicates for each measurement. Statistical analysis was performed by the Mann–Whitney test, comparing treated samples versus untreated proteins (^*^ = *p* < 0.01, ^**^ = *p* < 0.01, ^***^ = *p* < 0.001, n.s. = not significant); f,g) sequences and the sum of modifications across the determined peptide sequences (not determined sequences are specified and excluded) for MART, oxMART, PMEL, and oxPMEL; h) individual oxPTM numbers occurred in MART and oxMART as well as in i) PMEL and oxPMEL; j,k) a percentage of oxPTM that increased after exposure to cold plasma.

Therefore, for the first time, we oxidized melanoma antigens MART and PMEL using cold gas plasma technology and studied the immunological consequences in vitro and in vivo (**Figure** **1**a) in this work. OxPTMs were examined by mass spectrometry after cold plasma exposure, and protein size was measured using the dynamic light scattering method. Oxidized MART and PMEL proteins, as well as native counterparts, were used as vaccines to immunize C57BL/6 mice prior to challenge with B16F10 melanoma cells. In isolated tumors, changes in the TME, such as infiltrated immune cells and the secretory profile of pro‐ and anti‐inflammatory cytokines, were determined. Splenocytes of vaccinated mice were incubated ex vivo with proteins and tumor lysates to investigate lymphocyte proliferation and activity.

## 2 Result

### 2.1 Reactive Oxygen Species Induced Oxidative Post‐Translational Modification on MART and PMEL

Tumor‐associated antigens MART and PMEL were exposed to multiple reactive oxygen species, and possible modifications were investigated in oxidized MART (oxMART) and oxidized PMEL (oxPMEL) (Figure 1a,b). Interestingly, the correlation coefficient measured by dynamic light scattering did not show any effects of cold plasma on both proteins, suggesting no structural changes or higher molecular structures (Figure 1c; Figure S1a, Supporting Information). The fact that cold plasma did not lead to oligomerization or aggregation was confirmed by determining the protein's diameter. After cold plasma exposure, oxMART and oxPMEL did not show changes in the protein size (Figure 1d; Figure S1b, Supporting Information) and did not alter the polydispersity (Figure 1e). We further analyzed oxidative post‐translational modifications (oxPTM) on native and cold plasma‐treated proteins by liquid chromatography‐mass spectrometry (LC‐MS) after trypsin digestion. Herein, oxPTMs were identified in sequences of both native and oxidized proteins, but changes in oxPTM numbers were determined after cold plasma exposure (Figure 1f,g; Figure S1c,d, Supporting Information). Indeed, an increased number of oxidation (+1O), trioxidation (+3O), deamidation (‐1H‐1N+1O), and carbamylation (+1C+1H+1N+1O) were found in oxMART, when compared to untreated MART (Figure 1h; Figure S1e, Supporting Information). Still, most of the determined oxPTMs were found in both MART and oxMART. On the contrary, oxPMEL showed an overall greater difference to the native form (Figure 1i; Figure S1e, Supporting Information). For instance, decreased numbers were identified for oxidation, trioxidation, chlorination, nitration, and nitrosylation, but elevated levels were found for quinone, L‐Aspartic‐4‐semialdehyde, iminoacetronitrie + diydroxy, cyanocarbene + oxo, and formaldehyde. When comparing additional oxPTMs in MART and PMEL, individual modifications were found for both proteins with different proportions in the total number, and more individual oxPTMs were found in oxPMEL (Figure 1j,k; Figure S1f,g, Supporting Information). However, investigating individual amino acids, we identified additional oxPTM in oxMART and a shift of modifications in oxPMEL (Figures S2 and S3, Supporting Information). For instance, glutamine on position 29 (Q29) was oxidized in oxPMEL but not in PMEL, whereas amino acids at positions 43, 84, 149, 155, and 182 were oxidized in PMEL but not in oxPMEL. We next examined whether these oxPTMs alter the cellular response to the protein due to changes in immunogenicity, leading to immune cell activation, proliferation, and differentiation.

### 2.2 Tumor Overcome Immunization Following Vaccination with TAA and oxTAA

**Figure 2:**
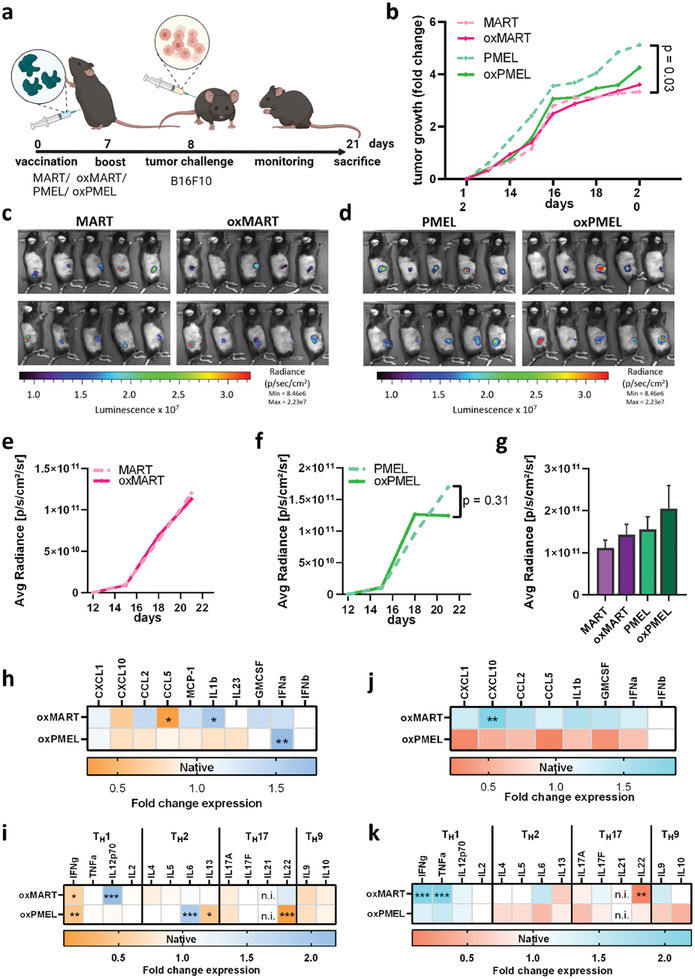
Vaccination of wildtype C57BL/6 mice with oxTAA promoted an anti‐tumor immune response. a) wild‐type C57BL/6 were vaccinated twice i.p. with 10 µg of either MART, oxMART, PMEL, or oxPMEL followed by subcutaneous inoculation of luciferase‐expressing B16F10 melanoma cells; b) mean tumor growth in each group; c,d) representative images of luciferin's luminescent detection in vivo in anesthetized mice and radiance over time to determine tumor growth after e) MART or oxMART immunization and f) PMEL or oxPMEL vaccination; g) final luminescence signal in slightly older mice, before mice were euthanized and tumors isolated; cytokine analysis of explanted tumors after enzymatic digestion to measure inflammatory state in (h) young and j) slightly older mice; T_H_ cell cytokine profile (T_H_1/T_H_2/T_H_17/T_H_9 segmentation) in (i) young and k) slightly older mice. Data in b, e, f, and g are shown as mean or mean ± SEM from 4–9 mice. Statistical analysis was performed by the Mann–Whitney test, comparing treated samples versus untreated proteins (^*^ = *p* < 0.01, ^**^ = *p* < 0.01, ^***^ = *p* < 0.001, n.s. = not significant). Data in (h–k) are mean values of 4–8 technical replicates measuring pooled supernatants of ten mice. Statistical analysis was performed by an ordinary two‐way anova (^*^ = *p* < 0.01, ^**^ = *p* < 0.01, ^***^ = *p* < 0.001, n.s. = not significant).

Oxidation‐mediated changes in immunogenicity of MART and PMEL were investigated by performing a gold standard assay of in vivo rechallenging experiment. C57BL/6 mice were administered intraperitoneally with native (MART/PMEL) or oxidized proteins (oxMART/oxPMEL) (**Figure** **2**a). Two days after a boost injection, luciferase‐expressing melanoma cells were injected, and tumor growth was measured and analyzed dynamically. The tumors measured over the test period show that tumor growth in MART‐immunized mice is slower than in mice that received a PMEL vaccination (Figure 2b; Figure S4a–d, Supporting Information). Differences between PMEL versus oxPMEL are small, and MART versus oxMART shows almost no change in tumor growth. The bioluminescence‐based target variable's evaluation using in vivo bioluminescence imaging confirmed this observation (Figure 2c,d). On the last day of measurement, luciferase signals in MART and oxMART immunized mice were similar (Figure 2e). In contrast, oxPMEL‐vaccinated mice showed lower luciferase signals when compared to mice that received PMEL (Figure 2f). In addition, the gene expression of luciferase was reduced in tumors after oxPMEL vaccine (Figure S4e, Supporting Information). Interestingly, immunizing slightly older mice (6–8 weeks of age) with oxPMEL and oxMART slightly increased the luciferase signal (Figure 2g). Ten days after injecting B16F10 melanoma cells, mice were euthanized, and tumors were isolated to analyze the TME. Therefore, tumors were enzymatically digested before cytokine concentration and immune cell infiltrates in the tissue were determined via flow cytometry. Interestingly, individual changes in inflammation‐related cytokine expression were observed in mice that received oxidized MART and PMEL compared to their native counterparts (Figure 2h; Figure S4f, Supporting Information). For instance, interleukin (IL)1b was significantly increased in oxMART‐vaccinated mice, and oxPMEL led to a reduction of IL1b. Similar effects were observed for cytotoxic T‐cell‐secreted chemokine CCL2. Both oxTAA vaccines elevated interferon (IFN)α, which modulates the TME and promotes an antitumor immune response. OxMART and oxPMEL individually changed the concentrations of other cytokines important for T_H_ subset polarization in the TME (Figure 2i). IL12p70 (T_H_1) was increased after vaccination with oxMART but not after oxPMEL injection. On the contrary, oxPMEL triggered IL6 (T_H_2) secretion and reduced IL13 and IL22 expression. In slightly older mice, no changes were found after oxPMEL vaccination, and only minor changes in cytokine secretion were observed after oxMART vaccination (Figure 2j,k). Still, after oxMART vaccination, the IFNγ, CXCL10, TNFα, and IL‐22 levels were in contrast to secretion in young mice.

### 2.3 OxMART and oxPMEL Vaccine Altered Intratumoral Cytotoxic T‐Cell Response

**Figure 3:**
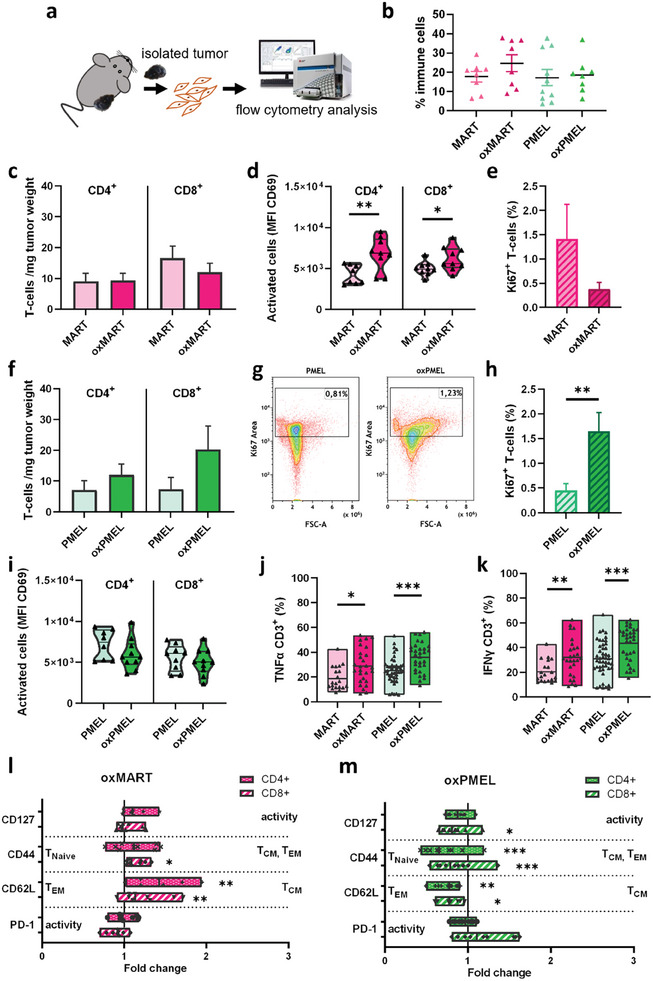
OxMART vaccine elevated T‐cell activity and oxPMEL triggered T‐cell proliferation. a) study workflow showing flow cytometry analysis of separated cells in isolated tumors; b) a percentage of tumor‐infiltrated CD45^+^CD3^+^ and CD45^+^IAIE^+^ immune cells; CD4^+^ helper cells and cytotoxic CD8^+^ in tumors after c) MART, oxMART vaccination or f) PMEL, oxPMEL vaccination; d) increasing mean fluorescent intensity (MFI) of CD69 representing T‐cell activation in tumors after MART/ oxMART vaccination, and i) no changes after PMEL, oxPMEL vaccination, respectively; e) percentage of proliferating intratumoral T‐cells indicated by positive Ki67 signal is reduced after oxMART vaccination; g) representative dot plot and h) quantified signals of increasing Ki67 signal in tumors of oxPMEL vaccinated mice; j,k) intracellular cytokines are elevated in CD3^+^ cells in tumors of oxTAA vaccinated mice; analysis of CD4^+^ and CD8^+^ subpopulations reveal a l) positive shift to T_cm_ cells after oxMART vaccination and m) a oxPMEL driven shift of T_em_ differentiated cells. Data are shown as individual values or mean ± SEM from 5–10 mice. Statistical analysis was performed by the Mann‐Whitney test, comparing treated samples versus untreated proteins (^*^ = *p* < 0.01, ^**^ = *p* < 0.01, ^***^ = *p* < 0.001, n.s. = not significant).

We further separated cells from explanted tumor tissue and investigated immune cell subpopulation via flow cytometry (**Figure** **3**a). The percentage of tumor‐infiltrated immune cells (CD45^+^CD3^+^ and CD45^+^IAIE^+^ cells) in tumors of oxTAA‐vaccinated mice did not change when compared to tumors from TAA‐vaccinated mice (Figure 3b). Furthermore, oxMART‐vaccination did not change CD4^+^ or CD8^+^ T‐cell numbers (Figure 3c). Surface marker expression of activation marker CD69 was significantly elevated in CD8^+^ and CD4^+^ T‐cells in tumors from oxMART‐vaccinated mice (Figure 3d) and intracellular proliferation marker ki67 in CD3^+^ cells was decreased (Figure 3e). On the contrary, oxPMEL vaccination led to a slightly increased number of T‐cells per tumor mass, and proliferation marker ki67 was significantly increased (Figure 3f–h). The activity of CD8^+^ and CD4^+^ T‐cells was not affected in tumors from oxPMEL‐vaccinated mice when compared to PMEL‐vaccine (Figure 3i). In order to investigate which immune cells secreted the cytokines previously measured in the supernatants of lysed tumors, we measured intracellular cytokines using flow cytometry. Albeit IFNγ was decreased in tumor supernatants, intracellular staining in CD3^+^ cells revealed slightly increased IFNγ and TNFα signals after oxTAA injection (Figure 3j,k). Similar effects were observed in the IAIE^+^ population (Figure S5a,b, Supporting Information). However, increased cytokine secretion after oxTAA vaccination did not change the number of antigen‐presenting cells within the TME or the activity (Figure S5c,d, Supporting Information). Suggesting that the activation state and proliferation of lymphocytes correlate with T‐cell differentiation, the surface marker expression of CD44 and CD62L were investigated. Interestingly, in tumors of oxMART‐vaccinated mice, levels of CD8^+^62L^+^ central memory cells (T_CM_) increased, while oxPMEL led to elevated CD8^+^62L^−^ effector cell (T_EM_) subsets (Figure 3l,m). On the other hand, CD127 was reduced after oxPMEL vaccination when compared to tumors from PMEL‐vaccinated mice, suggesting less tumor‐infiltrated immunosuppressive regulatory T‐cells (T_regs_). In line with these findings, the amount of T_regs_ was probably not affected as FoxP3 and intracellular IL10 remain unchanged (Figure S5e–g, Supporting Information). All in all, narrowed effects were observed when comparing oxMART versus MART regarding oxPTMs, tumor‐infiltrated cells, and cytokine secretion, and oxPMEL showed stronger deviation from PMEL (Figure S5h,i, Supporting Information). To confirm the generation of memory T‐cell subsets, we next isolated splenocytes and incubated them ex vivo with native or oxidized TAA.

### 2.4 Ex Vivo Stimulation with Proteins Revealed a Narrowed T‐Cell Activation Rate After oxMART Vaccination and Increased Activation After oxPMEL Vaccination

**Figure 4:**
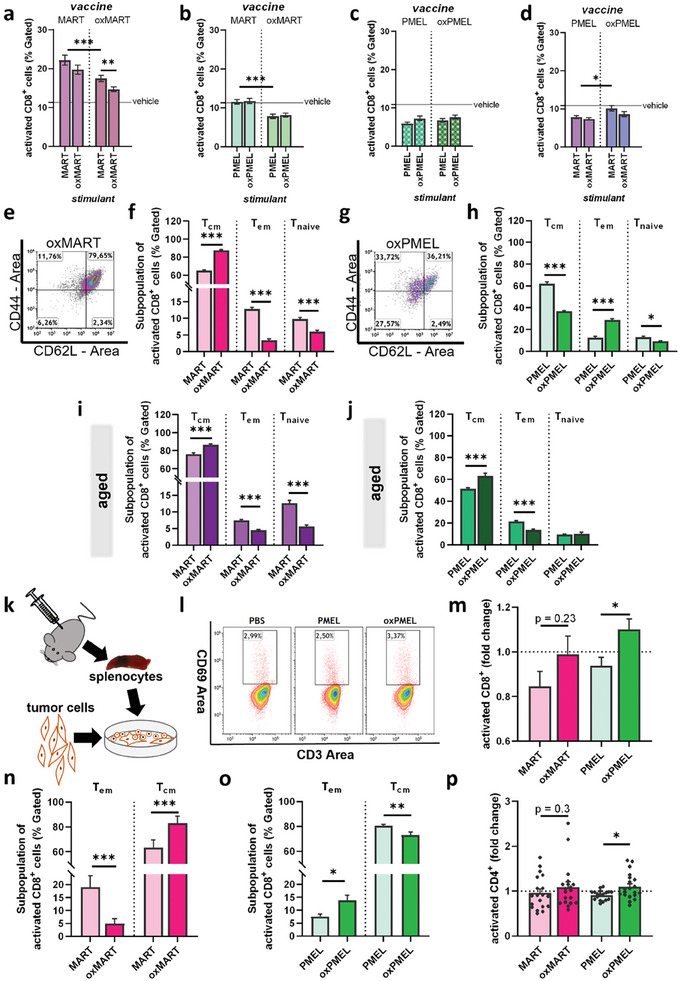
Restimulation with proteins and melanoma cells confirmed oxMART‐driven T_cm_ T‐cell differentiation and oxPMEL‐driven T_em_ T‐cell differentiation. CD8^+^ activity was determined in splenocytes from MART, oxMART vaccinated mice after stimulation with a) MART or oxMART, b) PMEL or oxPMEL; spleens from PMEL or oxPMEL vaccinated mice were stimulated with c) MART or oxMART, d) PMEL or oxPMEL; e–j) splenocytes of vaccinated mice were re‐stimulated with corresponding protein, e.g., splenocytes from oxMART vaccinated mice incubated with oxMART, and subopulation in activated cells was analyzed; e) representative dot plot of flow cytometry analysis showing and f) quantified majority of activated CD8^+^ T_cm_ cells in oxMART stimulated splenocytes from oxMART‐vaccinated mice; g) representative image and h) quantified shift of T‐cell differentiation with a majority in activated CD8^+^ T_em_ cells after incubating splenocytes from oxPMEL vaccinated mice with oxPMEL; i,j) aged mice were vaccinated with MART, oxMART, PMEL or oxPMEL, and isolated splenocytes were challenged with the vaccine, showing a majority of activated CD8^+^ T_cm_ cells; k) splenocytes from vaccinated, tumor‐bearing mice were incubated with viable melanoma cells; l) representative dot plot of IAIE^−^CD3^+^CD8^+^CD69^+^ splenocytes to quantify m) activitated T‐cells in B16F10 co‐cultured splenocytes from TAA, oxTAA vaccinated mice; subpopulation analysis show n) a majority in T_cm_ cells after incubating splenocytes from oxMART vaccinated mice with melanoma cells, and o) a majority in activated CD8^+^ T_em_ cells in splenocytes from oxPMEL vaccinated mice after stimulation; p) activitated CD4^+^ T‐cells in B16F10 co‐cultured splenocytes from TAA or oxTAA vaccinated mice. Data are shown as mean ± SEM or individual values of two technical replicates per spleen isolated from five mice per group; statistical analysis was performed using the Mann–Whitney test (^*^ *p* < 0.05; ^**^ *p* < 0.01; ^***^ *p* < 0.001).

The question that arose was whether oxidized protein vaccine inhibits or augments memory T‐cell subsets' activity. To this end, spleens of immunized, tumor‐challenged mice were isolated and enzymatically digested to get a single‐cell suspension. Separated splenocytes were incubated with native or cold plasma‐treated TAA for 24 h, and flow cytometry analysis revealed the level of activation and proliferation in different T‐cell subsets. Interestingly, splenocytes from MART and oxMART‐vaccinated mice showed an overall increased CD8^+^ T‐cell activation after stimulation with MART or oxMART ex vivo when compared to activation after incubation with PBS (**Figure** **4**a). However, splenocytes from oxMART‐vaccinated mice showed an overall reduced number of activated CD8^+^ T‐cells when compared to the effects of the MART vaccine. Furthermore, both vaccines (MART and oxMART) led to reduced activity in response to oxMART compared to an incubation with MART. We further incubated splenocytes from MART and oxMART‐vaccinated mice with PMEL and oxPMEL to investigate if oxMART vaccination led to reduced activity ex vivo. Indeed, the oxMART vaccine led to a lower number of activated CD8^+^ T‐cells compared to splenocytes from MART‐vaccinated mice (Figure 4b). PMEL and oxPMEL vaccination did not lead to any changes in the activation level of CD8^+^ after incubating splenocytes with PMEL or oxPMEL but was decreased when compared to vehicle (Figure 4c). However, oxPMEL vaccine led to significantly elevated CD8^+^ T‐cell activity after incubation with MART ex vivo, when compared to splenocytes from PMEL‐vaccinated mice (Figure 4d). We further investigated CD4^+^ T‐cell's activation. Similar to the CD8^+^ T‐cell response, the oxMART vaccine led to a drastic decrease in the total activation level, independent of the stimulant (Figure S6a, Supporting Information). On the contrary, the oxPMEL vaccine increased CD4^+^ activity in splenocytes after incubation with MART or PMEL (Figure S6b, Supporting Information). Furthermore, ex vivo incubation with oxMART led to slightly decreased activity, while oxPMEL showed an increased percentage of activated CD4^+^ cells when compared to incubation with the native protein. We next examined the phenotype of T‐cell subpopulations.

### 2.5 OxMART Vaccination Promoted CD8^+^ T_CM_ Response, and oxPMEL Polarized CD8^+^ T_EM_ Ex Vivo After Stimulation with Protein and Tumor Cells

Since the cell number of activated cells reached a significant level (>500 cells), we investigated the subpopulations using CD44 and CD62L to distinguish between effector memory, central memory, and naïve T‐cells in activated cells. Splenocytes incubated with their corresponding target protein, e.g., splenocytes from oxMART‐vaccinated mice incubated with oxMART, revealed a main population of central memory cells in activated CD8^+^ cells (Figure 4e). Interestingly, cells from oxMART‐vaccinated mice led to a population shift after stimulation with oxMART by significantly increasing the percentage of T_cm_ and decreasing both T_em_ and naïve populations compared to immune cells from MART‐vaccinated mice after stimulation with MART (Figure 4f). A similar trend of proportionate subpopulations, with the highest proportion of T_cm_, was found in both PMEL‐stimulated cells from PMEL‐vaccinated mice and oxPMEL‐stimulated splenocytes from oxPMEL‐vaccinated mice (Figure 4g,h). Again, the oxTAA vaccine led to a shift of the populations, but in the opposite direction to MART/ oxMART. A 20% reduction in the T_cm_ population was observed in oxPMEL condition, and the T_em_ population was three times higher than that of PMEL. For both protein vaccines and oxidized variants, the CD4^+^ cell population was not affected by stimulation with corresponding proteins (Figure S6c,d, Supporting Information). We also examined subpopulations after stimulation in splenocytes from slightly older mice after vaccination. In contrast to the previous results, similar effects on subpopulations of activated CD8^+^ cells were observed in mice of both age groups independent of the oxTAA vaccine (Figure 4i,j). Both conditions showed increased T_cm_ and reduced T_em_ populations after stimulation with the oxidized counterparts. Subpopulations of activated CD4^+^ T‐cell showed elevated T_em_ population and decreased amount of naïve cells in oxMART mice, whereas oxPMEL did not change the proportions of subpopulations (Figure S6e,f, Supporting Information).

To further investigate antigen‐mediated tumor‐specific T‐cell response, immune cells of vaccinated mice were co‐cultured with viable B16F10 cells for 24 h (Figure 4k). Interestingly, CD8^+^ T‐cells from oxPMEL‐vaccinated mice showed elevated activation levels compared to cells from PMEL‐vaccinated mice, and a similar trend was observed for oxMART‐vaccinated mice (Figure 4l,m). Again, we observed an increased amount of central memory phenotype CD8^+^ T‐cells in oxMART‐vaccinated mice, while the oxPMEL vaccine triggered an effector T‐cell response (Figure 4n,o). However, this activation did not lead to increased proliferation of CD8^+^ T‐cells in co‐culture with viable tumor cells (Figure S6g,h, Supporting Information). A slight increase in CD4^+^ activation was determined when splenocytes from oxTAA‐vaccinated mice were co‐cultured with tumor cells (Figure 4p). Elevated CD8^+^ and CD4^+^ activity correlated with increased secretion of the chemokine CCL2, IFNγ (in immune cells from oxPMEL vaccinated mice) (Figure S6i,j, Supporting Information).

## 3 Discussion

Antigen‐specific adaptive immune cells recognize TAA epitopes presented by MHC molecules to induce an anti‐tumor immune response. MHC ligands could reflect cancer‐associated oncogenesis pathways. However, 50–60% of source proteins are shared between cancer and non‐malignant cells, ≈13% of identified HLA ligands originated from overrepresented proteins (not necessarily mutation‐derived), and only a few tumor‐exclusive source proteins were alterations in cancer cells.^[^ ^37^ ^]^ Furthermore, due to the heterogeneity of tumors, tumor cells present variants of epitopes, such as mutated.^[^ ^38^ ^]^ or post‐translational modification.^[^ ^39^, ^40^ ^]^ antigens. These altered epitopes may be involved in immune escape mechanisms, so, we here investigated lymphocyte response to oxidized TAA.

Patient studies using melanoma‐specific TAA for therapeutic vaccination have been carried out since the 1990s.^[^ ^41^ ^]^ Still, recent results of the multicenter, double‐blinded, placebo‐controlled adaptive Phase III trial MAVIS (Melanoma Antigen Vaccine Immunotherapy Study) recommend further studies for optimization and using adjuvant or combinatorial treatments.^[^ ^42^ ^]^ It is also known that single proteins or multi‐peptide vaccines lead to the induction and proliferation of antigen‐specific T‐cells in experimental and clinical melanoma research,^[^ ^43^, ^44^, ^45^, ^46^ ^]^ partially yielding improved clinical outcomes.^[^ ^47^, ^48^ ^]^ However, aiming at antigen uptake by antigen‐presenting cells (APCs) for activation and maturation tending to activate lymphocytes, several adjuvant formulations, including mineral salts (e.g., alum), water‐in‐oil emulsion, Microorganism‐based adjuvants (e.g., CpG), and others, have been used for decades to immunize against pathogens or treat cancer, and allergies by enhancing host immune responses.^[^ ^49^, ^50^ ^]^ Intriguingly, RONS acts as a natural adjuvant, and RONS‐induced oxPTMs on epitopes may activate adaptive immune cells to promote an anti‐tumor immune response. Albeit the immunization experiment using oxidized TAA to enlarge the antigen pool failed, and none of the mice were able to clear the tumor after vaccination with TAA and oxTAA, we identified tumor‐suppressive characteristics in the tumor microenvironment that are similar for oxMART and oxPMEL. For instance, we found a higher density of tumor‐infiltrated CD8^+^, which is in line with previous studies, where increased amounts of CD8^+^, CD4^+^, and other immune cells in primary cutaneous melanoma correlated with prolonged survival from patients.^[^ ^51^, ^52^ ^]^ However, we did not monitor survival but slight tumor reduction after oxPMEL vaccination, and we suspect that stronger effects would have been observed with longer monitoring.

Interestingly, albeit tumor volume and luciferase signal did not change, vaccinated mice showed reduced Luc gene expression in isolated tumors, and oxPMEL vaccination showed significant reduction when compared to PMEL‐vaccinated mice. One could assume that Luc is recognized as another “foreign” antigen by immune cells, whereby the cells change their gene expression or are selectively attacked. Another hypothesis is that stromal cells in the tumor tissue increased, which express GAPDH, and this masked the qPCR results when normalizing luciferin expression. In other cancer cell types, there is evidence of stroma cells affecting cancer transcriptome analysis,^[^ ^53^ ^]^ as well as cancer proteomics results,^[^ ^54^ ^]^ which supports the second hypothesis. Nevertheless, the finished experiment provides insights into successful vaccination with anti‐tumor effects. Indeed, we determined elevated IFNα concentration in tumors of oxTAA‐vaccinated mice, a cytokine that also affects the general microenvironment of the tumor. Immunotherapy with IFNα correlates with tumor toxic effects in patients diagnosed with melanoma.^[^ ^55^, ^56^ ^]^ IFNγ, which was decreased in our study, is another cytokine relevant for direct tumor cell‐specific antitumor effects by, e.g., inhibition of proliferation, upregulation of MHC class I molecule expression, and thus enhancing their antigenicity – but also having contradicting effects (summarized elsewhere in refs. [57, 58]). Interestingly, IFNγ has been described to promote immunosuppressive mechanisms in melanoma cells, such as increasing PD‐L1 or recruitment of regulatory T‐cells T_regs_,^[^ ^59^ ^]^ and treatment of lung adenocarcinoma with IFNγ leads to enhanced expression of CD47 and increased tumor mass.^[^ ^60^ ^]^ However, we did not further investigate tumor cell characteristics, such as CD47, IDO, or PD‐L1 expression.

**Figure 5:**
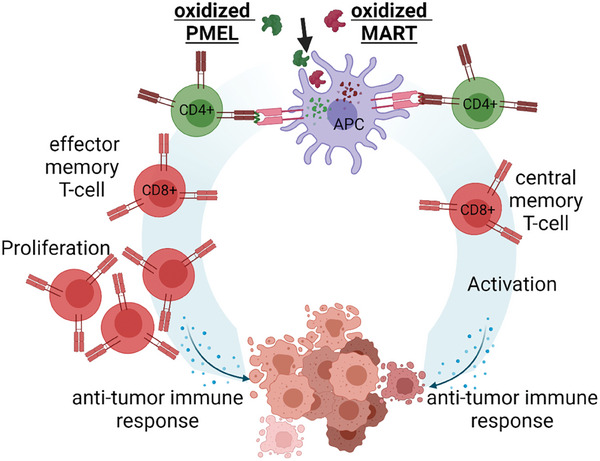
Hypothesis of two modes of action after vaccination with oxMART and oxPMEL.

The results discussed so far do not reveal any difference in the effect of the two TAAs examined, but we do see individual effects of different TAA. For instance, about half of the cytokines examined in tumors showed a similar concentration shift when mice received oxidized antigens, and the others were individual to the specific TAA. These include cytokines that promote a humoral immune response, another important aspect during an anti‐tumor immune response. Gilbert and colleagues determined that patient‐derived B‐cell cultures produce antibodies that recognize melanoma cells, followed by antibody‐mediated cellular cytotoxicity.^[^ ^61^ ^]^ We did not investigate the role of B‐cells in our experiments. We did not analyze antibodies targeting oxTAA, but further research should also be done to take this aspect into account and examine it. Antibodies directed toward oxidation‐specific epitopes are not uncommon, as they were determined in sera of patients diagnosed with rheumatoid arthritis,^[^ ^62^ ^]^ atherosclerosis, or diabetes.^[^ ^63^ ^]^ Interestingly, the binding affinity to cold plasma‐oxidized targets is altered when compared to native proteins.^[^ ^64^, ^65^ ^]^ (reviewed in ref. [66]). In the context of tumors, B‐cells promote CD8^+^ response to oxidized tumor lysate,^[^ ^14^ ^]^ and tumor‐associated B‐cells are important players in melanoma‐associated inflammation^[^ ^67^ ^]^ (reviewed in ref. [68]). In our study, we indeed examine increased CD8^+^ T‐cell activity after oxMART vaccination and oxPMEL vaccination promoted proliferation (**Figure** **5**). Oxidized proteins were shown previously to promote proliferative T‐cell activity.^[^ ^13^, ^69^, ^70^ ^]^ To our knowledge, these effects have not yet been investigated in cancers. OxPTMs play an important role in several diseases, e.g., ox‐induced dimerization of albumin was identified in patients diagnosed with chronic liver disease.^[^ ^71^ ^]^ and cirrhosis.^[^ ^72^ ^]^

To investigate oxPTM for the vaccine approach, we consciously decided against further adjuvants, such as LPS, Freund's adjuvants, or similar, because we wanted to examine the unfiltered effect of oxidations. One reason for making the modified antigen more recognizable to immune cells may be the fact that oxPTM creates danger signals that alert the immune system to the presence of a potentially harmful substance. Oxidation of antigens can lead to an altered structure and function,^[^ ^73^, ^74^, ^75^ ^]^ reviewed in,^[^ ^76^, ^77^ ^]^ and there is evidence of preferred uptake of HOCl‐modified proteins by antigen‐presenting cells.^[^ ^11^ ^]^ We previously screened several proteins for their capability to alter APC activity after oxidation.^[^ ^78^ ^]^ Based on the fact that each protein has its amino acid sequence for individual oxPTM, no overall conclusion could be made regarding the immunogenicity of oxidized proteins. This is consistent with the results presented here, where PMEL and MART show individual effects on T‐cells and, therefore, cannot be extrapolated to TAA from other cancers. It is important to mention that oxPTM for cellular mechanisms of protein uptake in APCs matters, as HOCl‐modified proteins get recognized via mannose receptor (CD206), scavenger receptors A (CD204), and CD36.^[^ ^79^ ^]^ In contrast, peroxynitrite‐modified proteins may be recognized by toll‐like‐receptor‐4 (TLR‐4), leading to the activation of pro‐inflammatory transcription factor NF‐κB.^[^ ^80^, ^81^ ^]^ In our study, we did not observe significant effects on tumor‐infiltrated IAIE^+^ cells or activity, so we did not investigate protein uptake by APCs.

Previously, we showed in a model system using ovalbumin (Ova), increased immunogenicity of cold plasma‐treated Ova by detecting preferred uptake of APCs, higher amounts of activated T‐cells, decreased tumor burden, and a broad set of oxPTMs.^[^ ^82^ ^]^ In the present study, we aim to confirm this effect for tumor‐relevant antigens for the vaccine approach. Our previous study suggested that oxidation and chlorination are paramount in the process of increasing immunogenicity. When evaluating chlorination and oxidations in TAA versus oxTAA, the following stand out: 1) chlorination was not determined in MART or oxMART, and oxPMEL showed reduced chlorination when compared to native PMEL; 2) oxidation (+1O) and trioxidation (+3O) were increased in MART after exposure to cold plasma, whereas these oxPTM were reduced in PMEL after treatment. However, in the last few years, we optimized our house‐designed database for oxPTM by expanding the database with additional, more accurate data. We, therefore, found enriched complex oxidations in oxPMEL, such as cyanocarben + oxidation or iminoacetonitrile/ + dihydroxy.

Based on the results of previous studies, it is suspected that significantly increased immunogenicity is induced by HOCl. In line with these findings, no chlorination was detected with either PMEL or MART, and no significant tumor‐reducing effects confirm this hypothesis. On the other hand, we observed altered T‐cell differentiation after oxMART and oxPMEL vaccination, which may be explained by the different oxidations found. For instance, oxidation and trioxidation were enriched in oxMART, whereas quinone and more complex oxidations were identified in oxPMEL. Still, it is questionable whether these modifications specifically influence the biological effects.

OxPTMs have altered immunogenicity, and besides acting as alarming molecules for APCs, oxPTMs on presented peptides on MHC may impact the ligand‐receptor interaction. The activation of a single T‐cell is not sufficient to induce an immune response, so a minimal number of cells must be present to overcome the threshold,^[^ ^83^, ^84^, ^85^ ^]^ and depending on the bond strength, activation is induced. Binding can be prevented or altered by modifications to the peptide, such as an amino acid substitution or post‐translational modifications (PTM) at the amino acid or structural level.^[^ ^86^, ^87^ ^]^ We hypothesize that oxPTM affects the position in the MHC complex's binding groove, similar to the prediction of neoepitope binding.^[^ ^88^ ^]^ Indeed, there is an affinity threshold for memory T‐cell differentiation following vaccination^[^ ^89^, ^90^ ^]^ that can be influenced by protein fold stability.^[^ ^91^ ^]^ and probably also by oxPTMs. However, further research needs to be done to confirm the different affinities of TCR and oxPTM‐derived MHC‐epitopes. Affinity can be important for the specificity of a TCR for the target. While we did not observe nonspecific T‐cell activation by oxidized antigens in the oxOva study,^[^ ^82^ ^]^ we here gathered interesting findings. Mice that were vaccinated with oxPMEL showed stronger T‐cell activity for the unknown MART protein than mice immunized with PMEL, but both were below the level of unstimulated cells. On the other hand, vaccination with oxMART led to decreased activity when cells were stimulated with native protein. These findings are in line with a previous study, where T‐cells specific for phosphorylated antigens were not activated when confronted with native protein.^[^ ^40^ ^]^ To further prove if oxTAA vaccination leads to the generation of oxTAA‐specific T‐cells, molecular mechanisms need to be investigated, which requires enrichment of these cells by, e.g., isolating T‐cells and expansion after stimuli with the protein. Then, e.g., TCR repertoire analysis or the adoptive transfer of these T‐cells into tumor‐bearing mice are necessary to conclude the generation of antigen‐specificity. However, it should be taken into account that it is not known exactly whether and, if so, which cellular mechanisms play a role in the recognition of oxidized antigens compared to the native protein. We, therefore, rule out a role of oxPTM interfering with TCR‐MHC‐I specificity and consider other, currently uncertain aspects, resulting in slightly reduced tumor mass after oxPMEL vaccination. Vaccine efficiency depends on the choice of the antigen(s) to be loaded onto DCs to increase T‐cell proliferation and activation, followed by reduced tumor mass. In fact, expanding the spectrum of different proteins, such as the combination of oxTAA proteins, would be a promising approach, as an efficient multi‐epitope vaccine using oxidized whole‐tumor lysates has previously been demonstrated.^[^ ^17^, ^35^, ^36^ ^]^

Conversely, our study provides evidence that individual oxidized proteins contribute to the T‐cell's activation, differentiation, and proliferation‐which, in turn, probably does not alone contribute to an efficient anti‐tumor immune response. As discussed before, APCs prefer to engulf oxidized proteins, and different oxPTMs lead to altered cellular mechanisms of protein uptake and signaling pathways that further impact immune responses and vaccine efficacies. Graciotti and colleagues showed that MHC‐II presented peptides (ligandome) that potentially lead to T‐cell activity change after feeding APCs with oxidized lysate.^[^ ^92^ ^]^ Considering the fact that RONS are present in the TME and oxidized antigens may impact tumorigenesis, treatment efficacy, and clinical outcome, more research needs to be done. Direct detection of some reactive species in patient samples is still difficult, although secondary effects can be detected more easily, such as long‐lived reactive species, identification of oxidized proteins, antioxidants, or RONS‐mediated markers. Altogether, oxidized proteins affect the recognition by immune cells, leading to different biological consequences, and more research needs to be done to correlate molecular effects and specific oxPTM.

## 4 Conclusion

Several oxidative post‐translational modifications (oxPTM) were found on MART and PMEL after cold plasma exposure without inducing dimerization or degradation. Immunization with oxidized MART or PMEL vaccines prior to challenge with viable melanoma cells correlated with significant changes in cytokine secretion and altered T‐cell differentiation of tumor‐infiltrated immune cells. OxPMEL vaccination increased T‐cell proliferation, and oxMART vaccination promoted activity in tumors. Restimulation of splenocytes with proteins and tumor lysates confirmed increased activation of central memory cells after oxMART vaccination, whereas oxPMEL promoted effector cell activity. Here, we present the first study that shows the molecular effects of oxidized melanoma‐associated antigens.

## 5 Experimental Section

### Protein Treatment with kINPen Plasma Jet

MART (BioVision, Milpitas, CA, USA) and PMEL (Cusabio, Houston, TX, USA) were reconstituted following the manufacturer's instructions, and treatment was carried out under the same experimental conditions as a pilot study using ovalbumin.^[^ ^82^ ^]^ For the treatment, proteins were freshly prepared and therefore diluted in PBS (100 µg mL^−1^), and 500 µL were pipetted per well of a 24‐well plate (Sarsted, Nümbrecht, Germany). Each well was treated for 1 min with the atmospheric pressure plasma jet kINPen IND (neoplasMed, Greifswald, Germany),^[^ ^93^ ^]^ using helium gas containing 2% oxygen (both Air Liquide, Paris, France; purity 99.9999%) as carrier gas. The gas ran at a flow rate of one standard liter per minute, and the distance between the nozzle and liquid was >2 mm to achieve a conductive effect.^[^ ^94^ ^]^ For control experiments, native proteins were treated with gas only (without ionization). After treatment, the evaporated volume was compensated by adding ddH_2_O.

### Photon Correlation Spectroscopy

Spectroscopy measurements of native or cold plasma‐treated MART and PMEL (100 µg mL^−1^) using a ZS90 dynamic light scattering (DLS) device (Malvern Instruments, Malvern, United Kingdom) equipped with a helium‐neon laser light source (632 nm). Proteins (material RI = 1.45, absorption = 0.001) in PBS (similar to water, dispersant RI = 1.33, viscosity = 0.954) were measured in low‐volume disposable cuvettes (ZEN0040). DLS measurements were done at a set angle of 90° and attenuator at 11. The size was measured at 22 °C, with an equilibration time of 120 s and cuvette position at 3 mm. Backscatter‐angled detection was performed at 173° with a scattering collection angle of 147.7°. Data analysis was carried out from four independent experiments measured in three replicates, each with several replicates having minimal time between repeats.

### High‐Resolution Mass Spectrometry

High‐resolution mass spectrometry coupled to liquid chromatography (LC/MS) was used to determine oxidative post‐translational modifications on TAA and oxTAA. The remaining samples injected into mice from two independent experiments were pooled, and MS measurement was performed in technical replicates using pooled samples. Protein vaccines (100 µg mL^−1^) were prepared for LC/MS analysis by trypsin digestion and buffer exchange (all chemicals of MS grade). After the determination of protein concentration using a fluoro‐spectrophotometer (DeNovix, Wilmington, DE, USA), 100 µg of protein was reduced in 250 mm triethylammonium bicarbonate buffer (TEAB; Sigma‐Aldrich, Taufkirchen, Germany) by adding 14.29 µg Tris(2‐carboxyethyl)phosphine (Merck, Darmstadt, Germany). After incubation at 60 °C for 45 min, 18.52 µg iodoacetamide (Merck, Darmstadt, Germany) was added, and samples were incubated for 20 min at 22 °C. Proteins were digested by trypsinization (Promega, Walldorf, Germany) for 18 h at room temperature and loaded on STAGE‐tips (Thermo Fisher Scientific, Dreieich, Germany). These were filled beforehand with three *Empore* discs (Sigma‐Aldrich, Taufkirchen, Germany), washed, and equilibrated with 100% methanol (Th Geyer, Berlin, Germany), 80% acetonitrile (ACN) (Th Geyer, Berlin, Germany)/ 0.5% formic acid (FA) (Merck, Darmstadt, Germany), 0.5% FA, 0.1% ACN, and 0.5% FA, respectively. Desalting was performed after loading the sample by washing three times with 0.5% FA and centrifugation (1200 × g, 1 min). To elute peptides from the STAGE‐tip, 50 µL 80% ACN/ 0.5% FA was added, and samples were centrifuged. Samples were dried using vacuum centrifugation and reconstituted in 20 µL 0.5% FA. LC/MS measurements were performed using a Q‐Exactive Orbitrap coupled to an UltiMate 3000 nano HPLC (both Thermo Fisher Scientific, Waltham, MA, USA). Peptides (100 ng) were loaded onto a PepMap C18 trap column. A Dionex UltiMate 3000 RSLCnano HPLC was connected to an Exploris 480 mass spectrometer with a Nanospray Flex ion source (ThermoFisher, Dreieich, Germany). Peptides were eluted from the trap column and separated on a 150 mm × 75 µm ID Acclaim PepMap C18 column using buffer A (0.1% v/v acetic acid) and buffer B (95:5 acetonitrile: 0.1% v/v acetic acid) at a flow rate of 300 nL min^−1^ with an elution gradient of 4–40% buffer B over 50 min. The column temperature was set to 40 °C. The mass spectrometer was operated in positive polarity mode with a transfer capillary temperature of 250 °C and a spray of 2 kV. Spectra were recorded in DDA acquisition mode (Top 15). Peptides were analyzed in full scan (350–1200 m/z, R = 120 000 at 200 m/z) with a target of 5 × 10^3^ ions, followed by 15 data‐dependent MS/MS scans with higher energy collisional dissociation (HCD, maximum injection time (IT) 50 ms, isolation width 1.0 m/z, NCE 30%), detected in orbitrap (R = 15 000 at 200 m/z). Dynamic exclusion was enabled and set to 30 s.

### MS Data Analysis

Raw LC‐MS/MS data were analyzed with Proteome Discoverer software, version 2.4.1.15 (ThermoFisher, Dreieich, Germany). MS/MS spectra were extracted from the raw files, and a search was performed using custom FASTA files, each containing a single protein sequence. SequestHT and MS Amanda 2.0 were used as search engines, and an in‐depth analysis of modifications was performed using the PMI‐Byonic plug‐in (Protein Metrics, Cupertino, CA, USA). The search engine parameters for PMI‐Byonic were set as follows: peptide mass tolerance = 10 ppm, fragment mass tolerance = 10 ppm, cleavage specificity = trypsin, missed cleavages = 2, and total common modifications = 2. A custom modification list was used based on previous experiments.^[^ ^30^ ^]^ (Figure S2, Supporting Information). The raw data were filtered, and modifications were considered valid if a protein modification occurred in both replicates of a group.

### Cell Culture

Melanoma cell line B16F10‐Luc was used for injection in mice after vaccination and experiments in vitro. Cells were cultured in plastic flasks (Sarstedt, Nümbrecht, Germany) with DMEM high glucose, containing 10% FCS, 1% penicillin/ streptomycin, 1% L‐Glutamine (PAN Biotech, Aidenbach, Germany) at 37 °C, 95% humidity, and 5% CO_2_. Every three days, cells were subcultured by washing them with PBS (PAN Biotech, Aidenbach, Germany), detaching with accutase (BioLegend, Amsterdam, Netherlands), washing after centrifugation and seeding 10% in a new flask with fresh media. For experiments, cells were detached and counted using flow cytometry (CYTOFLEX S; Beckman‐Coulter, Krefeld, Germany). Dead cells were excluded from the analysis by adding DAPI (Carl Roth, Karlsruhe, Germany).

### Animal Experiments

C57BL/6 mice, all female, 4–6 and 6–8 weeks (grouped as slightly older) of age, were commercially purchased (Charles River Laboratories, Sulzfeld, Germany) and kept in animal cages with a maximum of five mice per cage for 2 weeks before experiments started. Ethical approval was received from the local authority (Landesamt für Landwirtschaft, Lebensmittelsicherheit und Fischerei in the state of Mecklenburg‐Vorpommern, Germany; approval number M‐V 7221.3‐1‐022/19). C57BL/6 mice received intraperitoneal (i.p.) injections of 100 µL of PBS with or without 10 µg of MART, cold plasma‐treated MART (oxMART), PMEL, or cold plasma‐treated PMEL (oxPMEL). They received a boost vaccination (without adjuvant) 7 days later. On day 10, mice were challenged with a subcutaneous (s.c.) injection of 1 × 10^4^ B16F10‐Luc melanoma cells in Matrigel Matrix (Corning, Glendale, Arizona, USA). Every 2 days, mice were anesthetized with isoflurane, and in vivo imaging (IVIS Spectrum S5; PerkinElmer, Hamburg, Germany) was performed after i.p. injecting 100 µL PBS containing luciferin (30 mg mL^−1^). After experiments ended, mice were sacrificed, and lymphoid organs and tumors were removed. Splenocyte isolation and tumor digestion were performed using the splenocytes isolation kit and tumor dissociation kit, respectively, in an OctaMACS Dissociator device (Miltenyi Biotec, Bergisch Gladbach, Germany). One animal receiving oxMART vaccine was excluded from the study because no luciferin was measured during the experiment, and no tumor was found during the autopsy.

### TIL Analysis

Infiltrated immune cells were investigated by staining single‐cell suspension of tumors containing 1 × 10^6^ cells in PBS using the following antibodies (BioLegend, Amsterdam, The Netherlands): IAIE (APC‐fire 750, clone M5/114.15.2), CD80 (APC, clone 16‐10A1), CD86 (APC, clone GL‐1), CD45 (AF700, clone 30‐F11), CD62L (PE‐Dazzle, clone MEL‐14), CD127 (PE‐Cy7, clone A7R34), F4/80 (PE, clone BM8), CD26 (PE‐Cy7, clone H194.112), CD69 (PE, clone H1.2F3), CD40 (AF488, clone HM40‐3), CD279 (PerCP 5.5), CD3 (BV421, clone 17A2), CD8 (BV510, clone 53–6.7), CD11c (BV605, clone N418), CD4 (BV785, clone GK1.5), NK1.1 (BUV395, clone PK136), CD44 (BV496, clone IM7), MHC‐I (BUV661, clone M1/42) for cell characterization and iFluor maleimide 860 (ATT Bioquest, Pleasanton, CA, USA) for live‐dead discrimination was added. Cells were stained for 30 min at 37 °C.

### Intracellular Marker Analysis

Intracellular staining was performed in cells after fixation and permeabilization (BioLegend, Amsterdam, The Netherlands; according to manufacturer's instruction) using CD8 (PE, clone 53–6.7), FOXP3 (AF647), CD3 (AF700, clone 17A2), CD45 (APC‐fire 750, clone RA3‐6B2), IL10 (BV421, clone JES5‐16E3), TNFa (BV510, clone MP6‐XT22), Ki67 (BV605, clone 16A8), IAIE (BV785, clone M5/114.15.2), NK1.1 (BUV4395, clone PK136). Cells were stained for 30 min on ice.

### Immune Cell Profiling Following In Vitro Restimulation

Activity after restimulation assay was measured after collecting the cells in V‐bottom plates (Thermo Fisher Scientific, Waltham, Massachusetts, USA) and washed three times with cold FACS washing buffer (Miltenyi Biotec, Bergisch Gladbach, Germany). For live/dead discrimination, cells were stained with activated Caspase 3/7 detection reagent (Thermo Fisher Scientific, Waltham, Massachusetts, USA) and Fc‐block (BioLegend, Amsterdam, The Netherlands) at room temperature for 10 min followed by incubation with fluorescently conjugated monoclonal antibodies targeting CD62L (PE‐Dazzle, clone MEL‐14), CD44 (PerCP‐Cy5.5, clone IM7), CD4 (PE‐Cy7, clone L3T4), CD25 (APC, clone PC61), CD3 (Alexa Fluor 700, clone 17A2), CD69 (brilliant violet 421, clone H1.2F3), and CD8 (brilliant violet 510, clone 53–6.7). For T‐cell analysis, unwanted cells were gated out using a dump channel containing zombie‐NIR as well as CD45R (APC‐Fire 750, clone RA3‐6B2) and I‐A/I‐E (APC‐fire 750, clone M5/114.15.2) (all BioLegend, Amsterdam, The Netherlands). Cells were stained for 30 min on ice. After staining, cells were washed three times, and analysis was performed using CYTOFLEX S or CYTOFLEX LX instruments (both Beckman‐Coulter, Krefeld, Germany). Data analysis was conducted using Kaluza analysis software 2.1 (Beckman‐Coulter, Brea, California, USA).

### Restimulation of Splenocytes

After splenocyte isolation following the manufacturer's instruction (Miltenyi Biotech, Teterow, Germany), the single‐cell suspension was prepared with a fully supplemented RPMI culture medium. For protein stimulation experiments, 5 × 10^5^ cells per 90 µL of medium were seeded per well of a 96‐well U‐bottom plate (Sarstedt, Nümbrecht, Germany), and 10 µL PBS or PBS containing MART, oxMART, PMEL, or oxPMEL were added. Co‐culture experiments with melanoma cells were carried out by adding 100 µL splenocytes (5 × 10^5^ cells) to previously seeded 1 × 10^3^ viable tumor cells. After incubation for 24 h, flow cytometry experiments were performed as described above (both Beckman‐Coulter, Krefeld, Germany). For the proliferation experiment, splenocytes were labeled with carboxyfluorescein succinimidyl ester (CFSE, 2.5 µm; BioLegend, Amsterdam, The Netherlands) before adding to the tumor cells. Three days later, cells were collected, washed, and labeled with antibodies as described above to identify proliferating T‐cells via flow cytometry analysis.

### Cytokine Measurement

Supernatants of single‐cell suspension after separation and from stimulated cells in culture were collected and investigated by multiplex cytokine analysis according to the manufacturer's instructions (LegendPlex; BioLegend, Amsterdam, Netherlands). This multiplex is a bead‐based sandwich immunoassay and was measured using flow cytometry (CytoFLEX S; Beckman‐Coulter, Krefeld, Germany) targeting interferons, tumor necrosis factor‐alpha, interleukins, and chemokines. For quantification, data analysis software (Vigene Tech, Carlisle, MA, USA) was utilized. A separate standard curve was calculated using fifth‐degree polynomials for each analyte, with attention to the analytes' specific detection limits.

### RNA Isolation and Quantitative PCR Analysis

Single‐cell suspension from isolated tumors was taken for RNA isolation according to the manufacturer's instructions (StemCell Technologies, Vancouver, Canada). The 1 µg of RNA was transcribed into cDNA, and qPCR was conducted in duplicate using SYBR Green I Master (RocheDiagnostics, Mannheim, Germany). Specific primers were used to quantify luciferase expression (forward: CACCGTCGTA TTCGTGAGCA; reverse: AGTCGTACTCGTTGAAGCCG; BioTez, Berlin, Germany), and the housekeeping genes GAPDH was targeted as an internal control for normalization. Gene expression was analyzed using the 2^−∆∆Ct^ method.

### Statistical Analysis

Graphing and statistical analysis were carried out using *prism* 9 (GraphPad Software, San Diego, California, USA). A comparison of the two groups was made using the Mann–Whitney test. A comparison of more than two groups was made using a one‐way analysis of variances (ANOVA). A comparison of more than two groups across different data sets was made using two‐way ANOVA. Levels of significance were indicated as follows: α = 0.05 (*), α = 0.01 (**), α = 0.001 (***).

## Conflict of Interest

The authors declare no conflict of interest.

## Author Contributions

R.C. and S.B. performed conceptualization and visualization. R.C., L.M., E.F., and S.B. performed the methodology. R.C. and L.M. performed software and validation. R.C. performed the formal analysis and data curation of MS results, and wrote the original manuscript draft. R.C., S.B., T.W., K.‐D.W. wrote, reviewed, and edited. S.B. performed supervision and project administration. All authors have read and agreed to the submitted version of the manuscript.

## Supporting information

Supporting Information

## Data Availability

The data that support the findings of this study are available from the corresponding author upon reasonable request.
