# Supplementary material for: Oxidized Melanoma Antigens Promote Activation and Proliferation of Cytotoxic T‐Cell Subpopulations
Source: Adv Sci (Weinh). 2024 Jul 3;11(33):2404131. doi: 10.1002/advs.202404131 (PMC11434111; doi:10.1002/advs.202404131)
Supplement: Supplementary file 1 — Supporting Information [file ADVS-11-2404131-s001.pdf]

ADVANCED SCIENCE
Open Access

Supporting Information

for *Adv. Sci.*, DOI 10.1002/advs.202404131

Oxidized Melanoma Antigens Promote Activation and Proliferation of Cytotoxic T-Cell Subpopulations

*Ramona Clemen, Lea Miebach, Debora Singer, Eric Freund, Thomas von Woedtke, Klaus-Dieter Weltmann and Sander Bekeschus**

Oxidized melanoma antigens promote activation and proliferation of cytotoxic T-cell subpopulations

Ramona Clemen[1], Lea Miebach[1], Debora Singer[1,2], Eric Freund[1,3], Thomas von Woedtke[1,4], Klaus-Dieter Weltmann[1], *Sander Bekeschus[1,2]

1 ZIK *plasmatis*, Leibniz Institute for Plasma Science and Technology (INP), Felix-Hausdorff-Str. 2, 17489 Greifswald, Germany

2 Department of Dermatology and Venerology, Rostock University Medical Center, Strempelstr. 13, 18057 Rostock, Germany

3 Department of Neurosurgery, Wien University Medical Center, Vienna, 1090 Austria

4 Institute for Hygiene and Environmental Medicine, Greifswald University Medical Center, Ferdinand-Sauerbruch-Str., 17475 Greifswald, Germany

* correspondence: sander.bekeschus@med.uni-rostock.de

Keywords: CAP; gas plasma technology; neoantigens; oxPTM; plasma medicine; reactive oxygen species; ROS; tumor-associated antigens

Supplemental Figure Legends

Figure S1. Oxidative post-translational modified melanoma-associated antigens MART and PMEL. Quantified area under the curve (AUC) of (**a**) correlation coefficient and (**b**) protein size. Data are mean values that include all individual data points from four independent experiments with three technical replicates for each measurement. Statistical analysis was performed by the Mann-Whitney test, comparing treated samples versus untreated proteins (* = p<0.01, ** = p<0.01, *** = p<0.001, n.s. = not significant); (**c,d**) number of oxPTM in amino acid sequences of MART, oxMART, PMEL, oxPMEL in identified fragments (blue); (**e**) differences of individual oxPTMs in plasma-treated proteins, compared to native proteins; additional occurring oxPTMs and their amino acid positions in (**f**) oxMART, and (**g**) oxPMEL.

Figure S2. Sequence and detected oxPTM in oxMART. The amino acid sequence of the MART protein and oxPTMs that are only present in oxMART (red, X) or present in MART and oxMART (orange, B).

Figure S3. Sequence and detected oxPTM in oxPMEL. Sequence of PMEL protein and oxPTMs that are individual in PMEL (blue, N), oxPMEL (red, X), occur in PMEL and oxPMEL (orange, B).

Figure S4. Mice vaccinated with oxTAA and challenged with viable melanoma cells. Tumor growth was measured via caliper; individual tumor size in mice that received (**a**) PMEL, (**b**) oxPMEL, (**c**) MART, or (**d**) oxMART vaccine; (**e**) after euthanizing mice on the final day, tumors were explanted and RNA was isolated to determine luciferase expression; (**f**) individual cytokine concentrations in tumors (from normalized values in figure 2h, i). Statistical analysis was performed by the Mann-Whitney test, comparing oxTAA versus TAA samples (* = p<0.01, ** = p<0.01, *** = p<0.001).

Figure S5. Intratumoral immune response. Tumors were isolated and digested for further analysis. (**a, b**) intracellular staining of cytokines revealed increased TNFα and IFNγ secretion in antigen-presenting cells in oxMART vaccinated mice; (**c**) number of antigen-presenting cells and (**d**) activation level was not affected; (**e, f**) regulatory T-cell cytokine IL10 and (**g**) intracellular T_{reg} marker FoxP3 was not altered; (**h**) OxPTMs were correlated with cytokine secretion, infiltrated immune cells, activity and proliferation of TiL. Principle component analysis to determine overall differences in MART, oxMART, PMEL and oxPMEL and (b) loading of the different parameters. Statistical analysis was performed by the Mann-Whitney test, comparing oxTAA versus TAA samples (* = p<0.01, ** = p<0.01, *** = p<0.001).

Figure S6. Ex vivo restimulation. $CD4^+$ activity was measured in splenocytes from MART, oxMART vaccinated mice after stimulated with (**a**) MART or oxMART, PMEL or oxPMEL; spleens from PMEL or oxPMEL vaccinated mice were stimulated with (**b**) MART or oxMART, PMEL or oxPMEL; subset in activated $CD4^+$ T-cells shows no shift of individual populations after oxTAA vaccination in (**c, d**) young mice or (**e, f**) aged mice; (**g**) representative histogram of CFSE signals and (**h**) quantified signal for proliferation in cells after stimulation with viable tumor cells. (**I, j**) cytokine secretion in supernatants of melanoma splenocyte co-culture. Data are shown as mean, mean ± SEM, or individual values of two technical replicates per spleen isolated from five mice per group; statistical analysis was performed using the Mann-Whitney test, comparing oxTAA versus TAA samples (* p<0.05; ** p<0.01; *** p<0.001).

Supplemental Figures

Figure S1

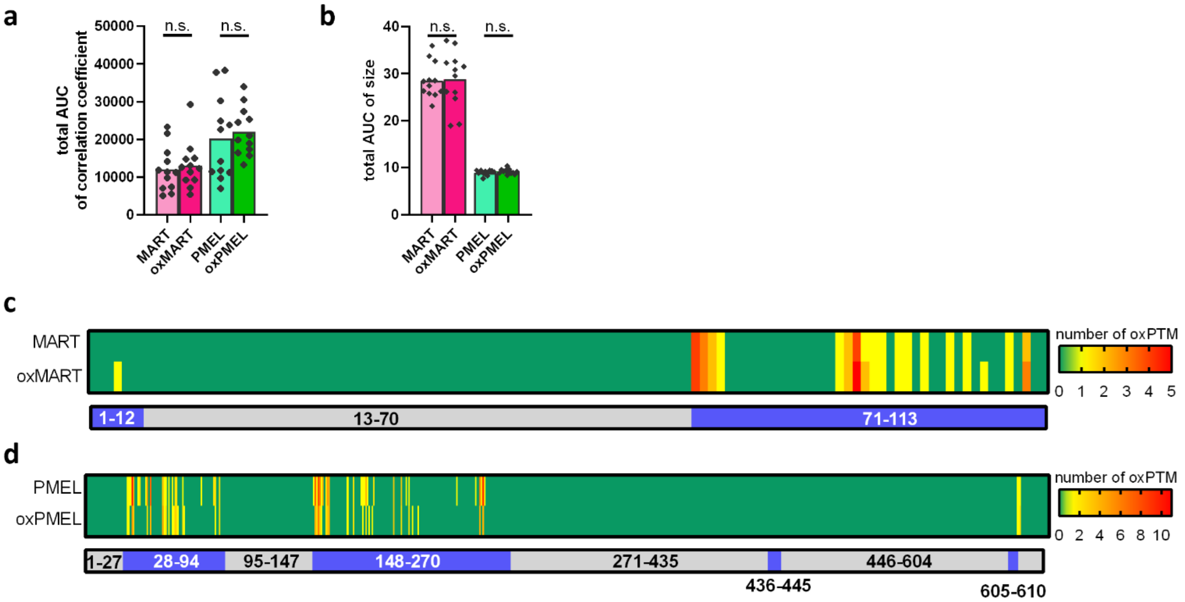

e

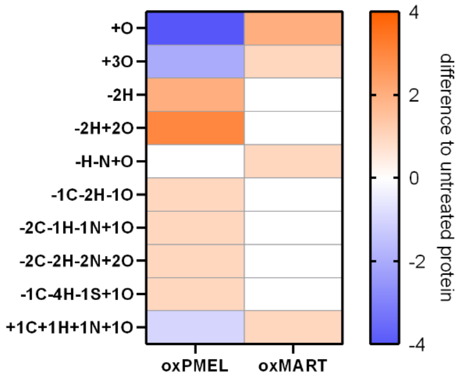

f

MART

position	aa	oxPTM
4	E	+1O
91	H	+1O
92	Q	-1H-1N+1O
106	S	+1O
111	Y	+3O

g

PMEL

position	aa	oxPTM
29	Q	+1O
31	W	+3O, -2H+2O
32	L	-2H+1O
51	W	-4H+2O
53	E	+1C+1H+1N+1O
58	N	-3H-1N+1O
59	C	-2H+2O
60	W	-2H+2Cl, -2H+2N+4O
61	R	+2O
64	Q	-1H-1N+1O
65	V	-2H
151	V	-2H
158	Y	-2H+1O
159	W	-2H
183	M	-1C-4H-1S+1O
185	V	-2H+2O
187	V	-2H+2O
201	H	-2C-1H-1N+1O, -2C-2H-2N+2O
206	F	-1H+1N+3O
211	Q	-1H+1Cl
213	P	-1C-2H-1O
217	S	-2H+1O
257	Y	-2H+1O
608	V	-2H+3O

Figure S2

		+1O	+2O	+3O	-2H	-4H	-1S	-1S+1O	-2H-1S	-2H+1O	-2H+2O	+1C+1H+1N+1O	+1C+1H+1N+2O	-1H+1N+1O	-1H+1N+3O	-1H-1N+1O	-3H-1N+1O	-1C-2H-1O	-2C-1H-1N+1O	-2C-2H-2N+2O
1	M																			
2	P																			
3	Q																			
4	E											X								
5	D																			
6	I																			
7	H																			
8	F																			
9	G																			
10	Y																			
11	P																			
12	R																			
[illegible]	[illegible]																			
71	R																			
72	C						B	B	B					B						
73	S					B				B	B									
74	C									B				B						
75	E	B																		
76	S																			
77	P																			
78	D																			
79	H																			
80	Q																			
81	D																			
82	S																			
83	R																			
84	L																			
85	S																			
86	S																			
87	Q																			
88	E																			
89	K											B								
90	S	B				B														
91	H	X	B										B						B	B
92	Q				B											X				
93	P																	B		
94	V				B															
95	V																			
96	P									B										
97	N									B										
98	A																			
99	P																	B		
100	P																			
101	A																			
102	Y														B					
103	E																			
104	K																B			
105	L																			
106	S	X																		
107	S																			
108	P																			
109	P				B															
110	P																			
111	Y	B	B	X																
112	S																			
113	P																			

Figure S3

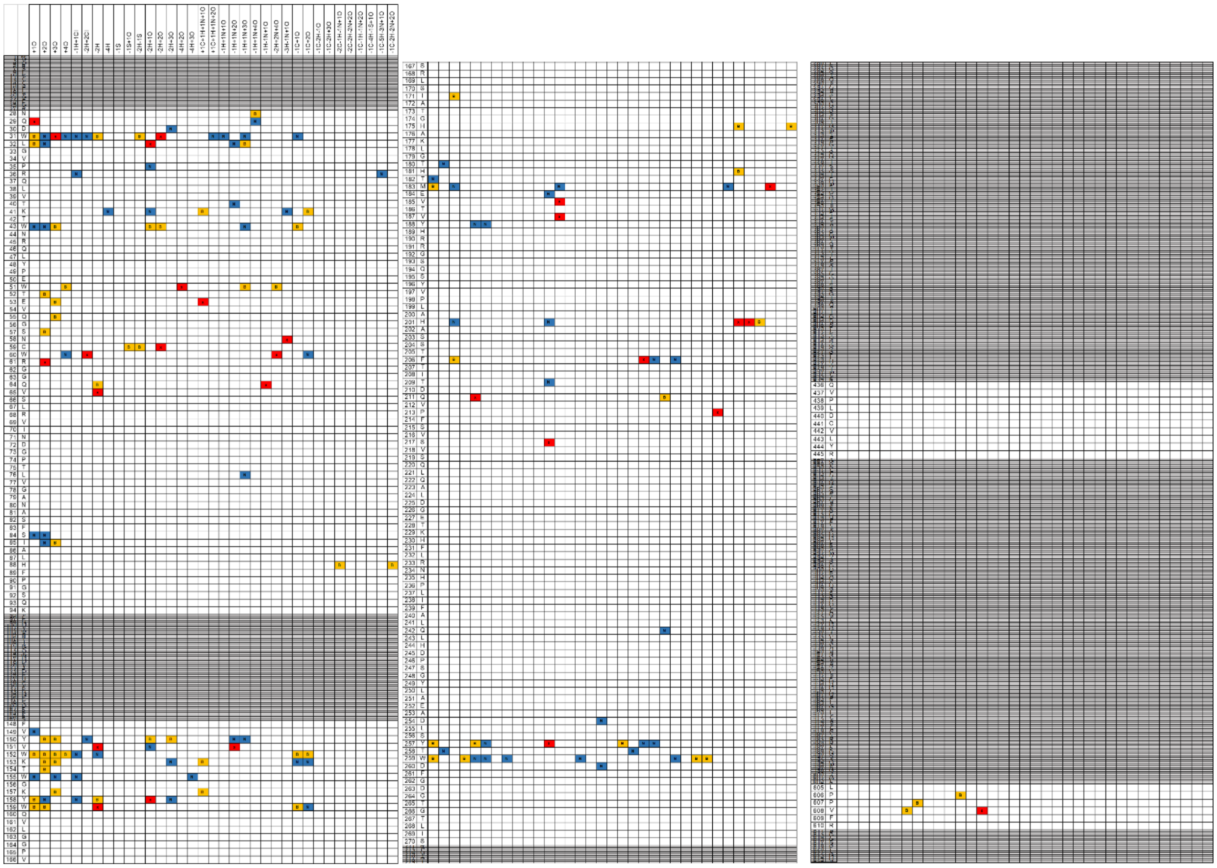

Figure S4

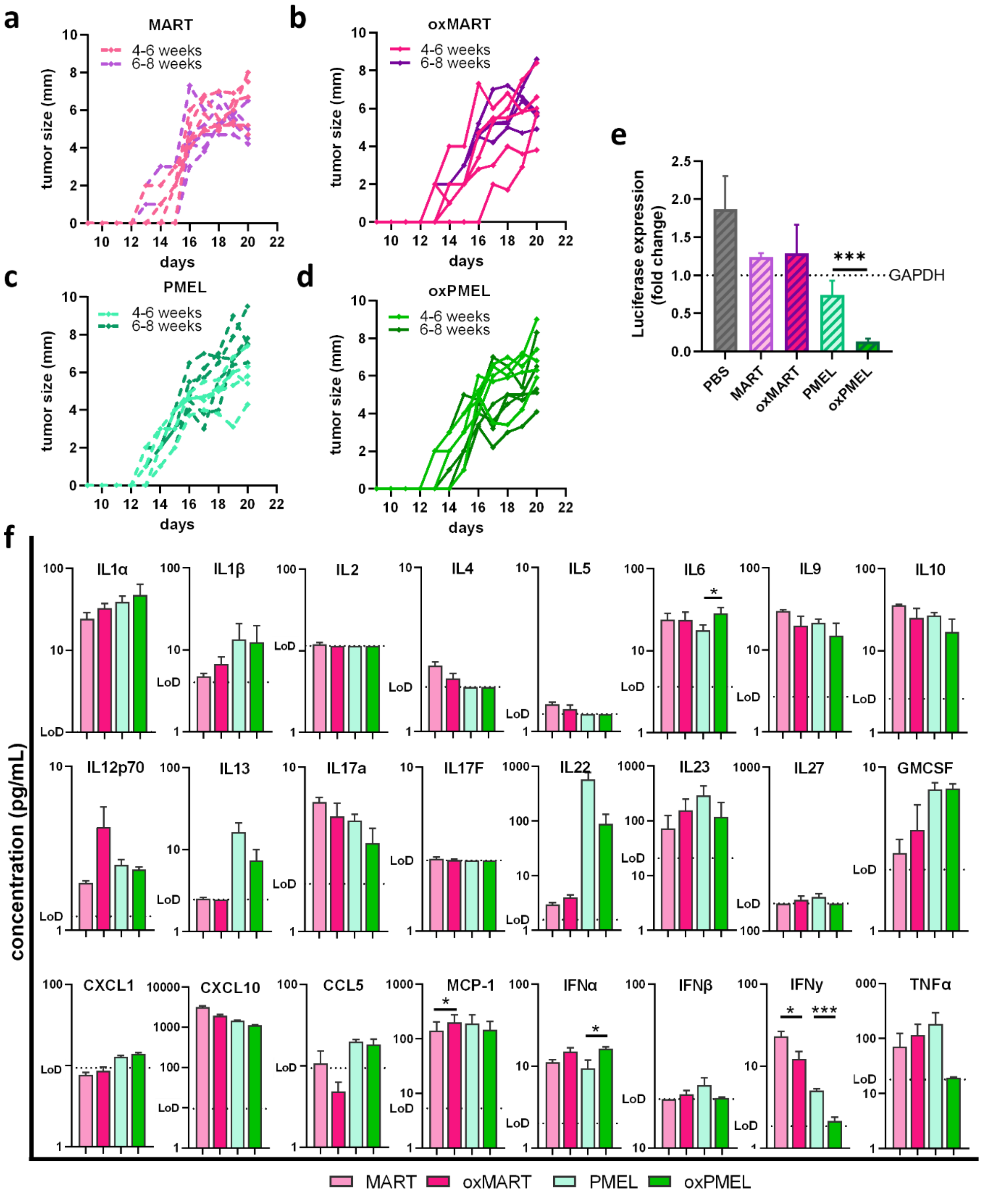
a
MART
4-6 weeks
6-8 weeks
tumor size (mm)
days
b
oxMART
c
PMEL
d
oxPMEL
e
Luciferase expression (fold change)
GAPDH
***
PBS
MART
oxMART
PMEL
oxPMEL
f
concentration (pg/mL)
IL1α
IL1β
IL2
IL4
IL5
IL6
IL9
IL10
IL12p70
IL13
IL17a
IL17F
IL22
IL23
IL27
GMCSF
CXCL1
CXCL10
CCL5
MCP-1
IFNα
IFNβ
IFNγ
TNFα
LoD
MART
oxMART
PMEL
oxPMEL

Figure S5

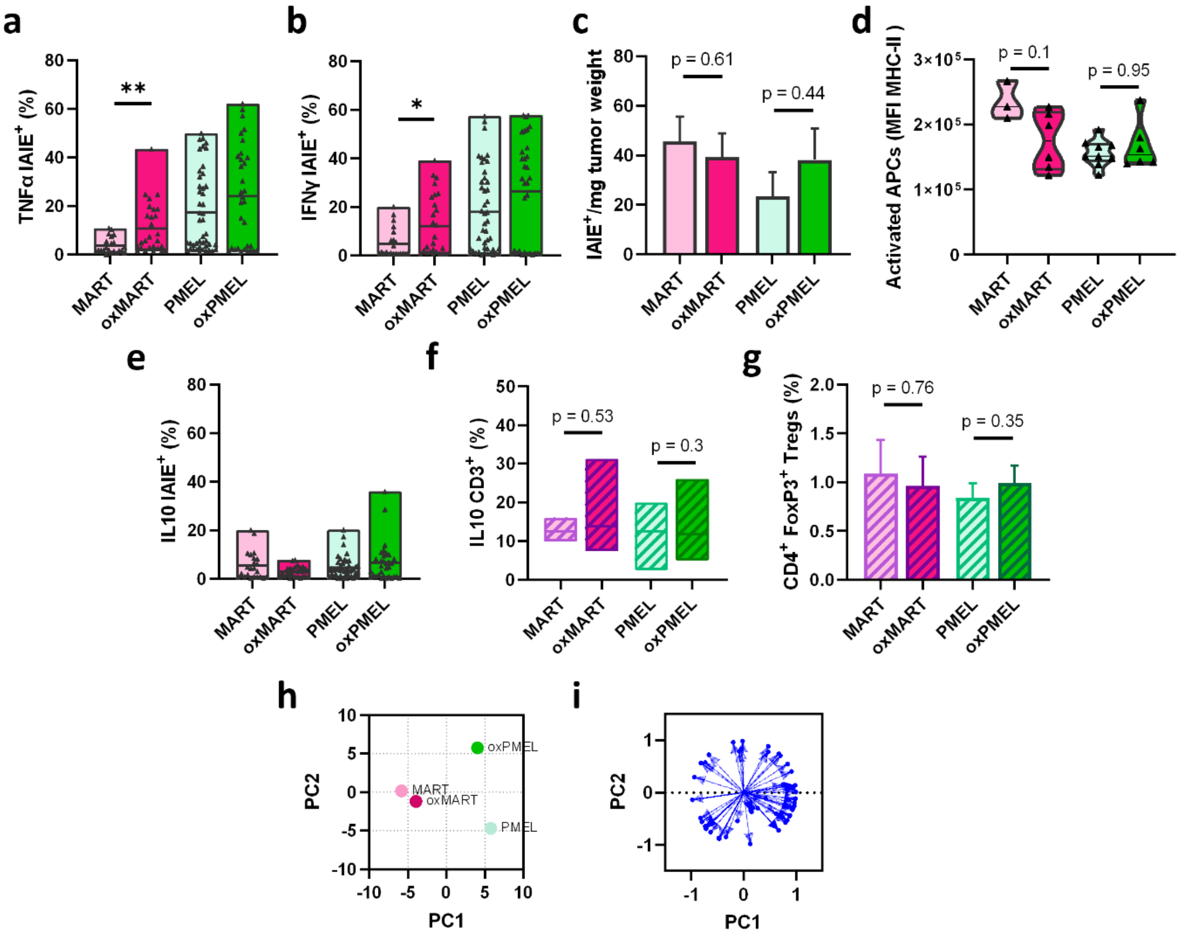

a
TNFα IAIE+ (%)
**
b
IFNγ IAIE+ (%)
*
c
IAIE+/mg tumor weight
p = 0.61
p = 0.44
d
Activated APCs (MFI MHC-II)
p = 0.1
p = 0.95
e
IL10 IAIE+ (%)
f
IL10 CD3+ (%)
p = 0.53
p = 0.3
g
CD4+ FoxP3+ Tregs (%)
p = 0.76
p = 0.35
MART
oxMART
PMEL
oxPMEL
h
PC2
PC1
i
PC2
PC1

Figure S6

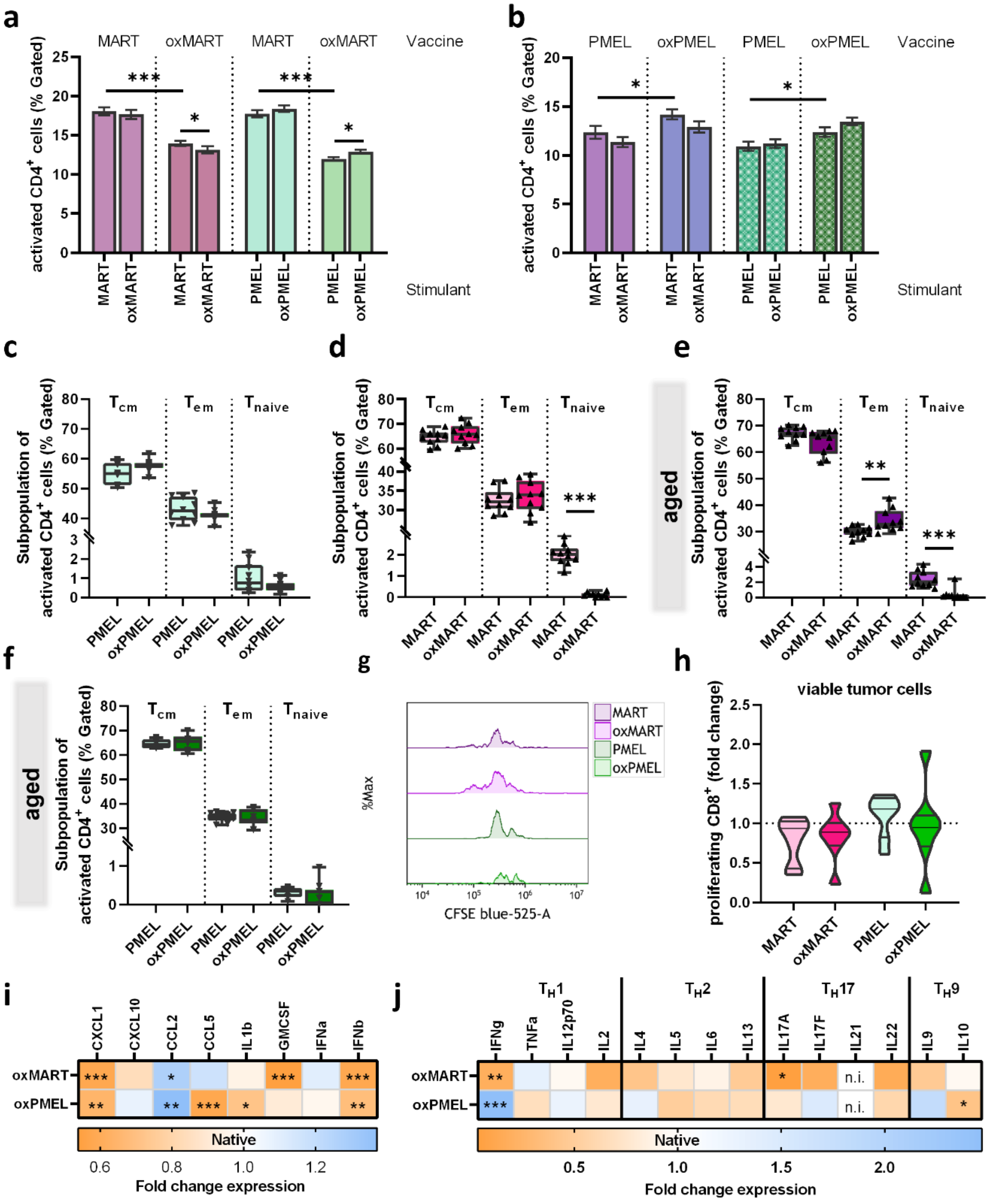

a
b
activated CD4+ cells (% Gated)
MART
oxMART
PMEL
oxPMEL
Vaccine
Stimulant
c
d
e
f
g
h
aged
Subpopulation of activated CD4+ cells (% Gated)
Tcm
Tem
Tnaive
%Max
CFSE blue-525-A
viable tumor cells
proliferating CD8+ (fold change)
i
j
CXCL1
CXCL10
CCL2
CCL5
IL1b
GMCSF
IFNa
IFNb
TH1
TH2
TH17
TH9
IFNg
TNFa
IL12p70
IL2
IL4
IL5
IL6
IL13
IL17A
IL17F
IL21
IL22
IL9
IL10
n.i.
Native
Fold change expression
